# Medication adherence levels and differential use of mental-health services in the treatment of schizophrenia

**DOI:** 10.1186/1756-0500-2-6

**Published:** 2009-01-12

**Authors:** Haya Ascher-Svanum, Baojin Zhu, Douglas E Faries, Nicolas M Furiak, William Montgomery

**Affiliations:** 1 US Outcomes Research, Lilly Research Laboratories, Eli Lilly and Company, Indianapolis, IN, USA; 2 Medical Decision Modeling Inc., Indianapolis, IN, USA

## Abstract

**Background:**

Adherence to antipsychotics for schizophrenia is associated with favorable clinical outcomes. This study compared annual mental-health service utilization by recent medication adherence levels for patients treated for schizophrenia, and assessed whether adherence levels change from pre- to post-psychiatric hospitalization.

**Methods:**

We analyzed data from a large prospective, non-interventional study of patients treated for schizophrenia in the United States, conducted between 7/1997 and 9/2003. Detailed mental-health resource utilization was systematically abstracted from medical records and augmented with patients' self report. Medication possession ratio (MPR) with any antipsychotic in the 6 months prior to enrollment was used to categorize patients as: adherent (MPR ≥ 80%, N = 1758), partially adherent (MPR ≥ 60% < 80%, N = 36), or non-adherent (MPR < 60%, N = 216). Group comparisons employed propensity score-adjusted bootstrap re-sampling methods with 1000 iterations, adjusting for baseline patient demographic and clinical characteristics identified a priori.

**Results:**

Adherent patients had a lower rate of psychiatric hospitalization compared with partially adherent and non-adherent patients (p < 0.001) and were more likely than non-adherent to engage in group therapy, individual therapy, and medication management. Most patients (92.0%) who were adherent in the 6 months prior to hospital admission continued to be adherent 6 months following hospitalization. However, 75.0% of previously partially adherent became adherent, and 38.7% of previously non-adherent became adherent following hospitalization.

**Conclusion:**

Adherence is associated with lower utilization of acute care services and greater engagement in outpatient mental-health treatment. Adherence is a potentially dynamic phenomenon, which may improve, at least temporarily, following patients' psychiatric hospitalizations.

## Background

Schizophrenia is often a persistent, debilitating, and costly disorder requiring diverse healthcare resources to manage the illness. Although adherence with antipsychotic medications is critical for reducing the risk of relapse and enhancing long-term functional outcomes [1], non-adherence is typically found in about 40%–50% of patients with schizophrenia [2].

Non-adherence is associated with increased risk of relapse and hospitalization [3-7], the most expensive health service component in the treatment of schizophrenia [8]. While the link between non-adherence and psychiatric hospitalization is well documented, little is known about the relationships between adherence and utilization of other mental-health resources in the management of schizophrenia, such as emergency services, partial hospitalization, and various outpatient services. The complexities of the illness and the heterogeneity of mental-health outpatient services call for data-driven analyses characterizing the use of specific categories of mental-health resources [8]. Outpatient services, including individual therapy, psychosocial and rehabilitation group therapy, case management, and medication management, may be conceptualized as "expectable" healthcare resources because the management of schizophrenia requires comprehensive and continuous care. Lack of expected outpatient resource utilization may signal that a patient is not engaged in the treatment plan and may therefore be non-adherent with medication and at increased risk of relapse and hospitalization.

Recent research also suggests that adherence is not an "all or none" phenomenon as many patients appear to be partially adherent with treatment, failing to take their medications as prescribed and having gaps in medication intake [3,9,10]. Therefore, studies now often evaluate categories of adherence, including partial adherence, non-adherence and full adherence [5,6,9,10]. Furthermore, the stability of medication adherence over time has received sparse attention, as has the potential impact of hospitalization on medication adherence level following hospital discharge. Although adherence has often been assumed to be a stable patient characteristic, recent data suggest that adherence level is not fixed [6,10], with non-adherent patients displaying less stable adherence status compared with adherent patients [6].

To expand on previous research and provide new information about the relationship between adherence and resource utilization, a type of information that is especially relevant for policy makers and other mental-health decision makers, this study used data from a large, prospective, naturalistic, non-interventional multi-site study of patients treated in the United States for schizophrenia (US Schizophrenia Care and Assessment Program; US-SCAP) [6,11,12] to: (1) compare utilization of various types of mental-health services over a 1-year period by patients' recent (6 months prior to enrollment) level of adherence with antipsychotic medications; and (2) assess whether psychiatric hospitalization impacts subsequent adherence levels in usual care.

## Methods

Participants in US-SCAP (N = 2327), which was conducted between July 1997 and September 2003, were enrolled from large systems of care across the United States. Inclusion criteria were broad: schizophrenia, schizoaffective disorder, and/or schizophreniform disorder based on the *Diagnostic and Statistical Manual of Mental Disorders, Fourth Edition *(DSM-IV) criteria as noted in patients' medical records (no structured diagnostic interviews were conducted at study entry), and ≥ 18 years old. The study protocol was reviewed and approved by the Institutional Review Board (IRB) at each site, and written informed consent was obtained from all participants. Further details about US-SCAP can be found elsewhere [6,11,12]. Patient characteristics at enrollment were assessed using a structured interview, medical record information, and standard psychiatric rating scales, including the Positive and Negative Syndrome Scale (PANSS) [13], the Global Assessment of Functioning scale (GAF) [14], Simpson-Angus Scale for extrapyramidal side effects [15], Abnormal Involuntary Movement Scale (AIMS) [16], the Quality of Life Scale [17], and the SCAP-Health Questionnaire (SCAP-HQ), a health questionnaire developed and validated for the study [18].

Mental-health resource utilization during the 1 year following study enrollment was systematically abstracted from medical records by trained examiners, using an abstraction form developed for this study. Patient-reported use of emergency psychiatric services on the SCAP-HQ was also used to help identify use of emergency services if this medical record information was missing. Antipsychotic medication use and adherence levels were derived from prescription information in patients' medical records. Medication possession ratios (MPRs) for the 6 months prior to enrollment were computed. MPR was defined as the sum of days with medication, divided by the number of days in the observation period, 6 months prior to enrollment. Consistent with prior research [5,19], the MPR for each patient was categorized as: adherent (MPR ≥ 80%), partially adherent (MPR ≥ 60% < 80%), and non-adherent (MPR < 60%).

The current analysis used data of US-SCAP participants who had resource utilization information for the 1 year following enrollment and antipsychotic medication information for the 6 months prior to enrollment. For statistical analyses, χ^2 ^tests, ANOVA, and Kruskal-Wallis tests were used to test for overall differences across adherence levels in patient characteristics at enrollment, the mean number of service contacts, and the proportion of patients with any of the following resource-utilization types: inpatient psychiatric hospitalization, emergency service, psychiatrist contact ("medication management"), partial hospitalization, psychosocial group therapy, individual therapy, and case management/Assertive Community Treatment (ACT).

Group comparisons (adherent vs non-adherent, adherent vs partially adherent, partially adherent vs non-adherent) were performed using propensity score-adjusted bootstrap re-sampling methods with 1000 iterations, adjusting for baseline patient demographic and clinical characteristics identified a priori. Propensity score stratification was used to adjust for potential confounding factors not attributable to adherence status. Bootstrap resampling was used to allow for a non-parametric assessment of mean outcomes in resource utilization. The propensity score for each patient was estimated with a logistic regression model with the following apriori selected covariates: age, gender, race, illness duration, study site, comorbid substance use disorder, schizoaffective disorder, comorbid personality disorder, comorbid borderline intelligence or mental retardation, uninsured, inpatient status at enrollment, and time elapsed between start of US-SCAP enrollment and the start date of each patient's study year. The estimated propensity scores were grouped into 5 strata based on quintiles, and bootstrap resampling was performed in a balanced fashion within each group across the propensity score strata. For example, each bootstrap replication for the non-adherent group would include approximately 43 re-sampled observations from each of the 5 strata. Pair-wise group comparisons on resource utilization were reported if the overall differences across the 3 groups were significant (p < 0.05).

## Results

### Patient characteristics and adherence levels

Of 2327 US-SCAP participants, 2010 (86.4%) had healthcare resource utilization for 1 year after study enrollment and comprised the analytic sample.

Patient characteristics at enrollment are presented in Table 1. Most participants were outpatients, male, and in their early 40s. About one third were African American and a small proportion was uninsured. Patients were ill for 2 decades, on average, and mostly moderately ill (per PANSS scores), with an impoverished quality of life (per Quality of Life scores). There were moderate rates of comorbid substance abuse disorder, personality disorder, and borderline intelligence or mental retardation. Participants also manifested mild-to-moderate levels of extrapyramidal symptoms and involuntary body movements (per Simpson-Angus and AIMS scores).

**Table 1:** Baseline patient characteristics by level of medication adherence in the 6 months prior to enrollment (N = 2010)

**Characteristic**	**Adherent** **(n = 1758)**	**Partially adherent** **(n = 36)**	**Non-adherent** **(n = 216)**	**p-value**
Age at baseline, mean ± SD years	42.4 ± 11.0	38.5 ± 9.3	39.9 ± 11.6	0.007
Male, n (%)	1075 (61.2)	21 (58.3)	135 (62.5)	0.870
African American, n (%)	651 (37.2)	21 (58.3)	77 (36)	0.031
Schizoaffective diagnosis, n (%)	583 (33.2)	13 (36.1)	66 (30.6)	0.684
Illness duration, mean ± SD years	21.7 ± 11.4	19.7 ± 10.0	20.3 ± 12.7	0.136
Uninsured, n (%)	105 (6.1)	5 (13.9)	21 (10.0)	0.020
PANSS total score, mean ± SD	70.3 ± 18.6	72.2 ± 22.4	70.9 ± 17.1	0.593
Quality of Life Scale, total score, mean ± SD	60.0 ± 22.3	58.9 ± 21.6	61.7 ± 22.4	0.781
Hospitalized at enrollment, n (%)	52 (2.96)	1 (2.78)	23 (10.65)	< 0.001
Comorbid substance abuse disorder, n (%)	483 (27.47)	12 (33.33)	78 (36.11)	0.024
Comorbid personality disorder diagnosis, n (%)	268 (15.24)	2 (5.56)	36 (16.67)	0.228
Comorbid borderline intelligence/mental retardation, n (%)	176 (10.01)	5 (13.89)	13 (6.02)	0.118
Simpson-Angus total score, mean ± SD	4.34 ± 4.08	4.00 ± 4.17	4.15 ± 4.08	0.547
AIMS total score, mean ± SD	3.35 ± 4.13	2.94 ± 3.73	2.51 ± 3.14	0.137

SD = standard deviation; PANSS = Positive and Negative Syndrome Scale; AIMS = Abnormal Involuntary Movement Scale

During the 6 months prior to enrollment, most patients were deemed adherent (87.5%). The rest were non-adherent (10.7%) or partially adherent (1.8%). Patients who were adherent prior to enrollment were significantly older than partially or non-adherent. African-American patients and uninsured patients were more likely to be partially adherent than adherent or non-adherent. There was a > 3-fold higher rate of hospitalization at enrollment among non-adherent compared with partially adherent or adherent. There were also higher rates of comorbid substance abuse disorder among patients with lower adherence levels (Table 1).

### Utilization of acute care services

During the 1 year following enrollment, adherent patients in the 6 months prior to enrollment were significantly less likely to have a psychiatric hospitalization (17.1%) compared with partially adherent (30.6%) and non-adherent patients (29.6%) (p < 0.05) (Table 2). Adherent participants also had about half as many inpatient admissions (0.28, 0.64 and 0.55 [p < 0.05 adherent vs partially and vs non-adherent]), and fewer total days hospitalized (8.8 vs 32.0 and 17.6 days [p < 0.05 adherent vs partially adherent]) (Table 3). The lowest mean number of emergency service contacts was observed for adherent patients, with statistically significant differences between adherent and non-adherent (p < 0.05) (Table 3). There were no significant differences in partial hospitalization (proportion of patients or mean number of contacts) by level of adherence (Table 2 and Table 3).

**Table 2:** Proportions of patients using inpatient and outpatient healthcare resource components during the 1 year after study enrollment, by treatment adherence level at enrollment

**Resource component**	**Adherent** **(n = 1758)**	**Partially Adherent** **(n = 36)**	**Non-adherent** **(n = 216)**
Hospitalization^a^	17.1%	30.6%	29.6%
Emergency Services	20.6%	25.0%	26.9%
Partial Hospitalization	23.6%	30.6%	21.8%
Psychiatrist Contact^b^	90.8%	91.7%	64.8%
Psychosocial Group^c^	47.1%	47.2%	35.2%
Individual Therapy^c^	78.9%	72.2%	60.2%
Case Management/ACT	61.6%	52.8%	53.7%

ACT = Assertive Community Treatment

^a ^Overall p < 0.05; significant difference between adherent vs partially and non-adherent (p < 0.05)

^b ^Overall p < 0.05; significant difference between non-adherent vs adherent and partially adherent (p < 0.05)

^c ^Overall p <0.05; significant difference between adherent vs non-adherent (p < 0.05)

**Table 3:** Mean number of inpatient and outpatient healthcare resource components used during 1 year following study enrollment, by treatment adherence level at enrollment (mean ± SD)

**Resource component**	**Adherent** **(n = 1758)**	**Partially Adherent** **(n = 36)**	**Non-adherent** **(n = 216)**
Inpatient admission^a^	0.28 ± 0.8	0.64 ± 1.3	0.55 ± 1.1
Days hospitalized^b^	8.8 ± 38.9	32.0 ± 82.6	17.6 ± 56.2
Emergency services^c^	0.25 ± 0.5	0.33 ± 0.6	0.37 ± 0.6
Psychiatrist contact^a^	13.7 ± 13.4	13.2 ± 15.0	8.7 ± 13.4
Partial hospitalization	24.9 ± 56.8	34.5 ± 65.2	21.9 ± 53.8
Group therapy	20.2 ± 44.7	13.6 ± 31.7	16.0 ± 42.9
Individual therapy	15.0 ± 20.7	17.7 ± 18.4	12.8 ± 18.3
Case management/ACT	1.14 ± 1.03	1.06 ± 1.04	1.05 ± 1.05

ACT = Assertive Community Treatment

^a ^Overall p < 0.05; significant differences between adherent and non-adherent and between adherent and partially adherent (p < 0.05)

^b ^Overall p < 0.05; significant differences between adherent and partially adherent (p < 0.05)

^c ^Overall p < 0.05; significant differences between adherent and non-adherent adherent (p < 0.05)

### Utilization of non-acute care services

Participants who were adherent prior to enrollment were significantly more likely in the 1 year following enrollment to engage in outpatient treatment, including psychosocial group therapy, individual therapy, and medication management ("psychiatrist contact") than non-adherent patients (Table 2). The frequency in which these outpatient services were used did not significantly differ by adherence level (Table 3) with the exception of contacts with psychiatrists, which were significantly fewer for non-adherent compared to adherent and partially adherent patients.

### Change in adherence level after hospitalization

Among 315 patients who were hospitalized, 276 patients were adherent 6 months prior to hospitalization, 8 were partially adherent, and 31 were non-adherent. Most patients who were adherent 6 months before hospitalization were also adherent 6 months afterward (92.0%) (Table 4). Most patients who were partially adherent before being hospitalized were adherent afterward (75.0%), and some patients who were non-adherent before being hospitalized were adherent (38.7%) or partially adherent (9.7%) afterward.

**Table 4:** Adherence levels prior to and after psychiatric hospitalization

**Adherence Level 6 Months *Before *Hospitalization**	**Adherence Level 6 Months *After *Hospitalization**		
	**Adherent**	**Partially Adherent**	**Non-adherent**
	**(n = 272)**	**(n = 8)**	**(n = 35)**
Adherent (n = 276)	92.0%	1.5%	6.5%
Partially adherent (n = 8)	75.0%	12.5%	12.5%
Non-adherent (n = 31)	38.7%	9.7%	51.6%

## Discussion

In this large, prospective, naturalistic, non-interventional study of patients treated for schizophrenia across the United States, patients who were deemed adherent immediately before enrollment had enhanced treatment outcomes in the subsequent year. Adherent patients evidenced a significantly lower risk of psychiatric hospitalization and better engagement in outpatient mental health care. Further, patient adherence proved to be a dynamic, rather than a static, phenomenon that appeared to be influenced by the experience of psychiatric hospitalization. Although most patients who were adherent before hospitalization continued to be adherent afterward, a large proportion of patients who were previously partially adherent or non-adherent became adherent in the 6 months after hospital discharge.

Current findings show that recently adherent patients had about half as many psychiatric hospitalizations and 2 to 4 times fewer total days hospitalized compared with partially and non-adherent patients. These findings are consistent with previous research [3-7]. Furthermore, recently adherent patients were more likely to be engaged in various outpatient treatment than non-adherent patients, suggesting that increased outpatient contact may decrease the risk of medication non-adherence [20].

Most patients in this study (87.5%) were deemed adherent and the rate of partial or non-adherence (12.5%) was lower than the 40% to 50% non-adherence rates reported in some previous studies but was broadly consistent with data reported in some other studies [3,20] in which non-adherence rates ranged between 15% and 20%.

In the present study, treatment adherence was also found to change from the 6 months pre-hospitalization to the 6 months post hospitalization, suggesting that adherence is a dynamic phenomenon. The reasons for the observed changes are unclear, but may be driven by increased attention to patient's medication management by members of the treatment team (e.g., case manager, psychiatrist), thus increasing intensity of medication management efforts post discharge, and leading, indirectly, to increased patients' MPR. The increased adherence post hospital discharge may also reflect involuntary commitment (compulsory treatment orders) to outpatient treatment for some patients. However, this information was not captured in our study. Another potential, but less likely, explanation is that hospitalization reflects a "teachable moment" for the patient, thereby leading to increased adherence with medication post hospital discharge.

Current findings need to be evaluated in the context of the study limitation. First is the naturalistic nature of the study. Although generally considered a strength, the observational nature introduces selection biases. We utilized propensity scoring to statistically adjust for selection bias; however, such methods adjust only for known and measured confounds and cannot address the prospect that the results may be partly attributed to or altered by unknown or unmeasured group differences. Second, current findings may not be generalizable to patients with private insurance because public payers covered healthcare services for almost all US-SCAP participants [11]. Third, adherence was evaluated by prescription information in patients' medical records, which may not accurately reflect medication prescription fills/refills or actual medication-taking behavior. Finally, the subgroup of partially adherent patients was small (n = 36), thus potentially limiting the conclusiveness of findings in this group.

## Conclusion

In the usual treatment of schizophrenia patients, medication adherence is associated with a lower risk of psychiatric hospitalization and with greater engagement in outpatient mental-health treatment. Adherence also appears to be a dynamic phenomenon, which may improve, at least temporarily, following patients' psychiatric hospitalizations. Current findings underscore the clinical and economic value of medication adherence, the need to separately assess acute-care and non-acute care resource utilization, and the realization that partially adherent patients, not only the non-adherent patients, should be targeted for adherence-promoting interventions.

## Competing interests

H. Ascher-Svanum, B. Zhu, D.E. Faries, and W. Montgomery are employees of and minor shareholders (stocks/options) in Eli Lilly and Company. N. Furiak is an employee of Medical Decision Modeling Inc., a company that has research and development contracts with Eli Lilly and Company.

## Authors' contributions

All authors contributed to study conduct and design. DF oversaw study design. HA-S, BZ, DF, and WM acquired data. All authors interpreted data. HA-S prepared and revised the manuscript with editorial assistance from Robin LeWinter, PhD, and Stephen W. Gutkin, Rete Biomedical Communications Corp. (Wyckoff, NJ, USA) with revisions by all authors. All authors read and approved the final manuscript.
